# Medial pivot-based total knee arthroplasty achieves better clinical outcomes than posterior-stabilised total knee arthroplasty

**DOI:** 10.1007/s00167-022-07149-2

**Published:** 2022-09-12

**Authors:** Michitaka Kato, Hideki Warashina, Shingo Mitamura, Akito Kataoka

**Affiliations:** Nagoya Joint Replacement Orthopaedic Clinic, 7 Iponbashi, Takadaji, Kita-Nagoya, Aichi 481-0011 Japan

**Keywords:** Anteroposterior laxity, Cruciate substitute, Medial pivot, Posterior-stabilised, Total knee arthroplasty, Sagittal stability

## Abstract

**Purpose:**

Stability in the sagittal plane, particularly regarding anterior cruciate ligament compensation, and postoperative functionality and satisfaction remain issues in total knee arthroplasty. Therefore, this prospective study compared the clinical outcomes between medial-pivot-based and posterior-stabilised total knee arthroplasty based on anterior translation and clinical scores.

**Methods:**

To assess outcomes of total knee arthroplasty for varus osteoarthritis, the anterior translation distance of the tibia relative to the femur was measured at 30 and 60° of flexion using a KS measure Arthrometer at 6 months postoperatively. The 2011 Knee Society Score, Forgotten Joint Score, visual analogue scale for pain, and range of motion were assessed at 6 months and 1 year postoperatively. The correlations among each score, anterior translation distance, range of motion, and visual analogue scale score for pain were investigated.

**Results:**

The medial-pivot and posterior-stabilised groups comprised 70 and 51 patients, respectively. The medial-pivot group exhibited a significantly shorter anterior translation distance at 60° flexion than the posterior-stabilised group. Furthermore, the medial-pivot group achieved significantly better outcomes regarding the visual analogue scale for pain, 2011 Knee Society Score, and Forgotten Joint Score than the posterior-stabilised group. A significant negative correlation was observed between the anterior translation distance and the function score of the 2011 Knee Society Score, whereas a significant positive correlation was found between the anterior translation distance and flexion angle, and between the extension angle and score of the Forgotten Joint Score or 2011 Knee Society Score. Significant negative correlations were also found between the pain visual analogue scale and both the 2011 Knee Society Score and Forgotten Joint Score.

**Conclusion:**

In total knee arthroplasty for osteoarthritis, the medial-pivot group displayed a shorter anterior translation distance than the posterior-stabilised group at 6 months postoperatively. The visual analogue scale score for pain was also significantly lower in the medial-pivot group than that in the posterior-stabilised group at both 6 months and 1 year postoperatively. Because a correlation was observed between the anterior translation distance and the function score, medial-pivot-based total knee arthroplasty was considered to significantly improve postoperative function compared to posterior-stabilised total knee arthroplasty.

**Supplementary Information:**

The online version contains supplementary material available at 10.1007/s00167-022-07149-2.

## Introduction

Total knee arthroplasty (TKA) is one of the most successful orthopedic surgeries for alleviating knee pain and recovering function. However, patient satisfaction after TKA is inferior to that after total hip arthroplasty. Reasons for dissatisfaction with TKA include [8] a confounding effect of malalignment [1, 23], instability [34], poor range of motion (ROM) [23], and residual pain [8]. Various implant designs, including cruciate-retaining, posterior-stabilised (PS), cruciate-substitute (CS), mobile-bearing, medial-pivot (MP), and bilateral cruciate ligament preservation, have been proposed. The association between implant and articular surface designs and improved postoperative clinical outcomes is of great interest to surgeons. Although posterior stability of the tibia is considered in CR and PS designs, a few implant designs allow for postoperative retaining of anterior cruciate ligament (ACL) function, which impacts postoperative clinical outcomes [16].

In PS implants, a post-cam mechanism replaces posterior ligament function and induces rollback, which prevents posterior translation of the tibia and restores good ROM in flexion. However, ACL function is not fully compensated in the PS design. Moreover, in MP implants, the medial condyle acts as a deep dish with a high degree of constraint, similar to a ball-and-socket joint, and the medial side possesses a high degree of anterior–posterior (AP) stability. In contrast, the lateral dish is shallow, with limited constraints, making it easier to reproduce the kinematics of a normal knee wherein the femur rotates laterally and rolls back against the tibia during flexion. Although studies have compared several MP implants and PS or cruciate-retaining implants, the superiority of the former regarding AP joint laxity has not been demonstrated and remains controversial [3, 4, 6, 11–13, 17–19, 21, 22, 30, 32, 35].

Therefore, this study compared the clinical outcomes, including anterior translation of the tibia relative to the femur, post-TKA knee function, and patient satisfaction, between MP and PS implants. It was hypothesized that MP implants could achieve better AP stability and improve functional performance.

## Materials and methods

### Study design

This prospective comparative study recruited patients with primary varus knee osteoarthritis (OA) scheduled for TKA between January 2019 and May 2020. This study compared clinical outcomes between patients who underwent TKA using the Evolution^Ⓡ^ CS system (MicroPort Orthopedics Inc., Arlington, USA) for MP TKA and the Attune^Ⓡ^ PS mobile-bearing implant (DePuy, Warsaw, Indiana, USA) for PS TKA. One of the two experienced surgeons used MP-type implants, and the other used PS-type implants. Each surgeon identified patients suitable for surgery in the outpatient clinic and performed the surgeries. Patients who did not provide consent: could not understand the questionnaire; had other types of implants or diseases, such as valgus knees, rheumatoid arthritis or other inflammatory diseases, or post-traumatic OA; or had had previous knee operations were excluded. The investigations included preoperative radiography (Rosenberg, lateral, axial and full frontal), full-length computed tomography (CT) evaluation of the lower limb, and preoperative 2011 Knee Society Score (KSS) evaluation [31, 33]. For evaluations after TKA, KSS, and AP, stabilities were measured using a KS measure Arthrometer (SIGMAX MEDICAL, Tokyo, Japan) at 6 months postoperatively, and KSS and Forgotten Joint Scores (FJS) were evaluated at 1 year postoperatively [5, 25]. ROM, lower extremity muscle strength, and visual analogue scale (VAS) for pain were assessed preoperatively and at 3 months, 6 months, and 1 year postoperatively.

Demographic and preoperative data are listed in Table 1. There were no significant differences in age, height, weight, or body mass index between the two groups. Extension was significantly more restricted in the MP group than in the PS group, and the PS group contained significantly fewer males than the MP group. Moreover, preoperative KSS was similar between the groups.

**Table 1:** Demographic and preoperative data

Variables	MP (*N* = 70)	PS (*N* = 51)	*t *Test
	Mean ± SD	Mean ± SD	*P *values
Age (years)	74.7 ± 6.1	74.9 ± 5.7	0.83
Sex (M:F)	19:51	5:46	0.02*
Height (cm)	155.0 ± 7.7	153.0 ± 5.4	0.10
Weight (kg)	63.1 ± 10.1	59.5 ± 9.6	0.05
BMI (kg/m^2^)	26.3 ± 3.7	25.4 ± 3.6	0.19
ROM			
Flexion (°)	130.8 ± 13.2	131.3 ± 15.6	0.85
Extension (°)	−7.6 ± 8.4	−4.4 ± 5.0	0.02
VAS for pain (mm)	44.2 ± 25.5	49.2 ± 29.0	0.31
KSS			
I symptoms	13.4 ± 5.6	11.8 ± 5.7	0.12
II satisfaction	16.4 ± 6.1	17.7 ± 6.1	0.23
III expectations	13.4 ± 4.0	13.3 ± 2.2	0.87
IV [1] walking and standing	16.6 ± 8.0	15.6 ± 7.7	0.50
IV [2] standard activities	17.3 ± 6.2	16.6 ± 6.2	0.55
IV [3] advanced activities	8.4 ± 5.5	8.2 ± 5.1	0.82
IV [4] discretionary activities	7.9 ± 4.1	7.0 ± 3.7	0.20
IV functional activities	50.3 ± 19.4	48.1 ± 14.8	0.51
Objective	34.7 ± 11.5	33.5 ± 11.5	0.58
Alignment	−8.5 ± 7.1	−8.6 ± 6.9	0.92
ROM	21.2 ± 6.2	21.8 ± 7.5	0.67
Instability	21.8 ± 5.0	20.6 ± 5.4	0.21

*BMI* body mass index, *KSS* Knee Society Score, *MP* medial pivot, *PS* posterior-stabilised, *ROM* range of motion, *SD* standard deviation, *VAS* visual analogue scale

^*^Chi-squared test

### Procedures

Cefamezin, a broad-spectrum antibiotic, was administered on induction, and intravenous tranexamic acid was administered preoperatively. Prophylactic antibiotics were administered until 24 h after surgery. All surgeries were performed under spinal anaesthesia with a measured resection technique. The medial parapatellar approach was used for both MP and PS TKA, but the trivector approach was also an option for PS TKA. The patella was not replaced in some cases. All osteophytes were resected with minimal soft-tissue dissection, and the ACL and posterior cruciate ligament (PCL) were resected in all cases.

On preoperative 3D CT, the knee valgus angle was measured via the trans-epicondylar axis. Subsequently, an osteotomy of the distal femur was performed using an intramedullary rod. An osteotomy of the proximal tibia was also performed, using an intramedullary rod with the Akagi line [2] or the Whiteside line as a reference for the rotation axis. The tibial axis was determined using the proximal tibial surface after full extension, using the distal femoral anterior surface (parallel to the Whiteside line) in flexion as references. Subsequently, tibial osteotomy was performed 8–10 mm from the lateral articular surface. Following osteotomy, the tibia was placed in extension and a spacer block was placed between the osteotomies to ensure the possibility of full extension. Subsequently, the posterior condylar angle was measured by preoperative CT and the rotation of the femoral component was determined by reflecting the angle of the posterior condylar axis.

The tibia was osteotomised with a 3° posterior tibial slope for both MP TKA and PS TKA, with the medial flexion gap slightly tighter than the lateral gap. The tibial components were mobile-bearing for PS and fixed type for MP. The spacer thickness that permitted full extension without hyperextension was selected. The implant was then cemented in place. Subsequently, the bone marrow cavity was plugged, and the tibial keel insertion was cemented. Suction drains were not used during surgery. Postoperatively, all patients received a local cocktail injection for pain relief, as well as mechanical prophylaxis and low-dose aspirin to prevent deep vein thrombosis. Ambulation was initiated in the morning after surgery. Pain management with multimodal medication was continued for several weeks. The average duration of hospitalisation was 10 days. After discharge from the hospital, outpatient rehabilitation was continued once or twice per week until 1 year after surgery for patients whose ROM was expected to improve.

### Assessment of sagittal stability

At 6 months postoperatively, physiotherapists independently evaluated the anterior translation distance of the tibia relative to the femur with patients in the supine position at 30 and 60° of knee flexion using the KS measure Arthrometer. The loads used were 89, 111, and 133 N. The examiners were blinded to the study design and implant type. The anterior translation distance was numerically displayed on the digital display board of the KS measure Arthrometer. The anterior pull-out was measured in triplicate and the mean was used as the measured value. ‘Instability’ was assessed based on the KSS.

### Image assessment

Postoperative radiography and CT were used for imaging evaluation. In the 3D CT scan after TKA, the frontal aspect of the femur was defined as the line connecting the centre of the femoral head and the centre of the implant with respect to the mechanical axis, and the axis of rotation was set based on the tangent line between the medial and lateral condyles of the femoral component. The coronal femoral component angle (CFA) was also measured. Rotation of the tibia was determined based on the frontal aspect of the femoral component, and the mechanical axis was determined as the line connecting the centres of the knee–ankle joints. A frontal image was created, and the coronal tibial component angle (CTA) was measured. The hip–knee–ankle angle (HKAA) was obtained by subtracting 180° from the sum of the CFA and CTA. The posterior femoral condylar offset ratio and the posterior tibial slope angle were measured from lateral knee radiographs. The angle of rotation of the femoral component was measured using the CT axial image (internal rotation + , external rotation−).

### Institutional review board study approval

This study was performed in accordance with the principles of the Declaration of Helsinki. The in-hospital study was approved by the Ethics Committee of the Nagoya Orthopedic Joint Replacement Clinic Ethics Committee (Date: 13 November 2018; No. 201811001). Written informed consent was obtained from all participants before study initiation.

### Statistical analysis

Statistical analyses were performed using SPSS^®^ software (version 26.0; IBM Corp., NY, USA). Data are reported as means and standard deviations, and *p* < 0.05 was considered statistically significant. Furthermore, t tests were used to compare variables between the MP and PS groups. Levene’s test for equality of variance was used to determine significance. Pearson’s correlation coefficients between KS measure values or ROM and each parameter were used to examine correlations.

G^*^power version 3.1.9.7 was used to verify the sample size for correlation analyses. When the effect size was 0.25, the power was 0.804, α error probability was 0.05, and sample size was 121 for two-tailed tests. For the study data, if the correlation co-efficient exceeded 0.25, the sample size was considered to be sufficient, but if it was below 0.25, there was a high possibility of a beta error due to an insufficient sample size.

To assess intra-observer and inter-observer reproducibility in coronal alignment, the measurements of all patients were recorded twice by one examiner and once by three examiners on 15 knees randomly selected from the study group. The intraclass correlation coefficients (ICC) (1,1) and (2,1) were used to evaluate intra-observer and interobserver reproducibility, respectively, for objective assessment. The Landis–Koch scale [20] was used to interpret the ICC with the following scoring criteria: 0–0.20, slight correlation; 0.21–0.40, fair correlation; 0.41–0.60, moderate correlation; 0.61–0.80, substantial correlation; and 0.81–1.00, perfect correlation.

## Results

### Study cases

**Fig. 1:**
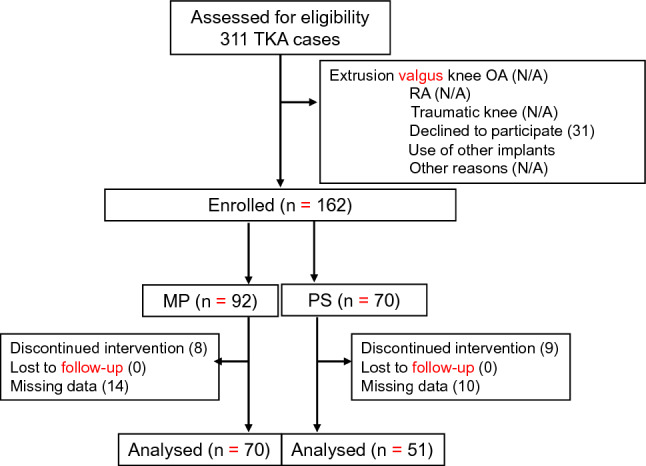
Study flowchart. *N/A* not assessed, *TKA* total knee arthroplasty, *RA* rheumatoid arthritis, *MP* medial pivot, *PS* posterior-stabilised

From January 2019 to May 2020, 92 MP and 70 PS cases were enrolled. Measurements of the anterior translation distance using the KS measure were attempted in all 162 cases, and complete data were obtained in 145 cases. Of these, 22 cases of MP and 19 cases of PS were excluded due to data unavailability or lack of routine postoperative visits due to the coronavirus pandemic. Finally, 70 MP and 51 PS cases were included (Fig. 1).

### Postoperative data

#### KS measure

KS measure values for anterior tibial translation are shown in Table 2. The translation distance was lower in the MP group than in the PS group, at both 30 and 60° of knee flexion. Additionally, significant differences were observed between the two groups for 89, 111, and 133 N at 60° of knee flexion.

**Table 2:** Means and standard deviations of the postoperative results based on *t* tests

Variables	MP (*N* = 70)	PS (*N* = 51)	*T* tests
	Mean ± SD	Mean ± SD	*P *values
KS-Measure 30° of knee flexion (mm)			
89 N	4.1 ± 2.2	4.5 ± 2.3	0.35
111 N	4.8 ± 2.5	5.4 ± 2.6	0.25
133 N	5.5 ± 2.7	6.1 ± 2.8	0.22
KS-Measure 60° of knee flexion (mm)			
89 N	3.6 ± 1.9	4.5 ± 1.7	** < 0.01**
111 N	4.1 ± 2.1	5.2 ± 1.8	** < 0.01**
133 N	4.6 ± 2.3	5.8 ± 1.9	** < 0.01**
ROM flexion			
3 months	121.8 ± 10.5	123.6 ± 15	0.43
6 months	124 ± 9.7	126.8 ± 8.3	0.10
1 year	125.2 ± 9.3	128.4 ± 10.2	0.07
ROM extension			
3 months	−2.9 ± 3.8	−3.6 ± 5.7	0.42
6 months	−1.5 ± 2.9	−2.9 ± 5.1	0.07
1 year	−1.1 ± 2.4	−2.4 ± 4.9	0.11
VAS for pain (mm)			
3 months	19.7 ± 19.4	22.8 ± 21.9	0.40
6 months	12.5 ± 19.5	22.8 ± 23.8	**0.01**
1 year	4.3 ± 8.5	15.6 ± 23.4	** < 0.01**
KSS 1 year			
I symptoms	21 ± 3.3	18.6 ± 4.9	** < 0.01**
II satisfaction	29 ± 7	25.3 ± 8	** < 0.01**
III expectations	10.3 ± 2.3	9.3 ± 2.6	**0.02**
IV [1] walking and standing	22.9 ± 6.4	21.2 ± 7.5	0.18
IV [2] standard activities	25.1 ± 4.4	22.6 ± 5.4	** < 0.01**
IV [3] advanced activities	15.4 ± 5.8	12.5 ± 6.6	**0.01**
IV [4] discretionary activities	11 ± 3.9	9.4 ± 4.6	**0.04**
IV functional activities	74.5 ± 16.1	65.7 ± 19.1	** < 0.01**
Alignment	24.5 ± 4.2	23.6 ± 6.9	0.39
ROM	24.2 ± 3.4	23.9 ± 4.4	0.68
Instability	22.6 ± 3.3	20.8 ± 3.9	** < 0.01**
FJS 1 year			
1 “Awareness in bed at night?”	1.1 ± 0.9	1.2 ± 1	0.43
2 “Awareness sitting on a chair for more than 1 h?”	1.1 ± 0.9	1.6 ± 1.1	**0.02**
3 “Awareness when you are walking for more than 15 min?”	1.1 ± 0.9	1.5 ± 1	**0.03**
4 “Awareness taking a bath/shower?”	0.9 ± 0.8	1.2 ± 0.9	0.06
5 “Awareness traveling in a car?”	0.9 ± 0.8	1.1 ± 0.9	0.33
6 “Awareness climbing stairs?”	1.5 ± 1.1	2 ± 1.1	**0.01**
7 “Awareness walking on uneven ground?”	1.7 ± 1.1	1.9 ± 1	0.21
8 “Awareness when standing up from a low-sitting position?”	1.9 ± 1	2 ± 1.2	0.55
9 “Awareness standing for long periods of time?”	1.6 ± 1	1.8 ± 1.1	0.3
10 “Awareness doing housework or gardening?”	1.6 ± 1	1.8 ± 1.1	0.33
11 “Awareness taking a walk/hiking?”	1.4 ± 1	1.9 ± 1.1	** < 0.01**
12 “Awareness doing your favourite sport?”	1.5 ± 1.1	1.9 ± 1.1	0.06
Total	66 ± 18.3	58.3 ± 20.4	**0.03**

Individual FJS questions were answered using a 5-point scale: never = 0 points, rarely = 1 point, almost never = 2 points, sometimes = 3 points, and almost always = 4 points

Cases with P<0.05 are shown in bold

*FJS* Forgotten Joint Score, *KSS* Knee Society Score, *MP* medial pivot, *PS* posterior stabilized, *ROM* range of motion, *SD* standard deviation, *VAS* visual analogue scale

#### ROM and pain VAS

The pain VAS was similar for both groups at 3 months postoperatively; however, the MP group reported significantly less pain than the PS group at 6 months and 1 year postoperatively (Table 2).

The postoperative knee flexion and extension were similar between both groups at 3, 6 months, and 1 year postoperatively (Table 2).

#### KSS

Six months postoperatively, the MP group exhibited significantly better performance in the KSS in terms of ‘Symptoms’, ‘Patient satisfaction’, ‘Patient expectations’, Functional activities, and Functional score subscales (including ‘Walking and standing’ and ‘Discretionary activities’), and the Objective knee indicator (‘Instability’). Moreover, at 1 year postoperatively, the MP group showed significantly better performance in the KSS regarding ‘Symptoms’, ‘Patient satisfaction’, ‘Patient expectations’, Functional activities, and Functional score subscales (including ‘Standard activities’ and ‘Advanced activities’), and the Objective knee indicator (‘Instability’) (Table 2).

#### FJS

At 6 months postoperatively, FJS2, FJS3, FJS4, FJS6, FJS7, FJS10, FJS11, and the overall score were significantly better in the MP group than in the PS group. Moreover, at 1 year postoperatively, significant differences were noted between the groups in FJS2, FJS3, FJS6, FJS11, and the overall score (Table 2).

### Correlations between KS measure values and different variables

#### ROM and pain VAS

The KS measure values and flexion angles were significantly positively correlated at 3, 6 months, and 1 year postoperatively for all measurement criteria. However, there were no correlations between KS measure values and extension angle or pain VAS (Table 3).

**Table 3:** Correlations between anterior tibial translation and ROM or clinical scores (only items with significant correlations)

	ROM flexion				KSS categories							FJS categories					
	Pre-op	Post-op 3 mos	Post-op 6 mos	Post-op 1 year	Pre-op	Post-op 6 mos		Post-op 1 year				Post-op 6 mos		Post-op 1 year			
					Instability	IV Discretionary activities	ROM	IV Walking and standing	IV Advanced activities	Functional activities	ROM	9	10	3	4	9	Total
KS 30°89 N																	
Correlation co-efficient	0.12	0.21	0.23	0.24	−0.07	−0.16	0.18	−0.18	−0.18	−0.15	0.19	0.19	0.22	0.26	0.24	0.18	−0.15
*P* value (two-sided)	0.20	**0.02**	**0.01**	** < 0.01**	0.45	0.08	**0.05**	**0.04**	**0.05**	0.10	**0.04**	**0.04**	**0.02**	** < 0.01**	** < 0.01**	0.05	0.10
KS 30° 111 N																	
Correlation co-efficient	0.12	0.21	0.24	0.24	−0.07	−0.16	0.18	−0.20	−0.19	−0.17	0.18	0.20	0.23	0.28	0.27	0.19	−0.17
* P* value (two-sided)	0.18	**0.02**	** < 0.01**	** < 0.01**	0.47	0.07	**0.05**	**0.03**	**0.04**	0.06	0.05	**0.03**	** < 0.01**	** < 0.01**	** < 0.01**	**0.04**	0.06
KS 30° 133 N																	
Correlation co-efficient	0.12	0.21	0.24	0.23	−0.08	−0.16	0.17	−0.21	−0.20	−0.19	0.17	0.21	0.24	0.28	0.27	0.20	−0.18
* P* value (two-sided)	0.18	**0.02**	** < 0.01**	**0.01**	0.40	0.09	0.06	**0.02**	**0.03**	**0.04**	0.06	**0.02**	** < 0.01**	** < 0.01**	** < 0.01**	**0.03**	**0.04**
KS 60° 89 N																	
Correlation co-efficient	0.18	0.32	0.35	0.37	−0.22	−0.30	0.17	−0.15	−0.17	−0.17	0.17	0.06	0.14	0.26	0.22	0.06	−0.11
* P* value (two-sided)	**0.05**	** < 0.01**	** < 0.01**	** < 0.01**	**0.02**	** < 0.01**	0.06	0.09	0.07	0.07	0.07	0.53	0.13	** < 0.01**	**0.02**	0.51	0.25
KS 60° 111 N																	
Correlation co-efficient	0.20	0.33	0.36	0.38	−0.23	−0.30	0.20	−0.15	−0.18	−0.17	0.20	0.06	0.15	0.25	0.23	0.06	−0.11
* P* value (two-sided)	**0.03**	** < 0.01**	** < 0.01**	** < 0.01**	**0.01**	** < 0.01**	**0.03**	0.11	0.05	0.06	**0.03**	0.52	0.10	** < 0.01**	** < 0.01**	0.53	0.23
KS 60° 133 N																	
Correlation co-efficient	0.19	0.33	0.36	0.38	−0.23	−0.29	0.22	−0.15	−0.20	−0.19	0.22	0.05	0.14	0.24	0.23	0.05	−0.10
* P* value (two-sided)	**0.04**	** < 0.01**	** < 0.01**	** < 0.01**	**0.01**	** < 0.01**	**0.01**	0.10	**0.03**	**0.04**	**0.02**	0.56	0.12	** < 0.01**	**0.01**	0.59	0.27

Cases with P<0.05 are shown in bold

*FJS* Forgotten Joint Score, *KSS* Knee Society Score, *mos* months, *Post-op* postoperative, *Pre-op* preoperative, *ROM* range of motion

#### KSS

At 6 months postoperatively, ‘Discretionary Activities’ (KSS Functional Score subscale) and KS measure values at 60° of knee flexion were significantly negatively correlated. At 1 year postoperatively, there were significant negative correlations between KS measure values and ‘Walking and Standing’ (KSS Functional Score subscale) at 30° of knee flexion, with loads of 89, 111, and 133 N. There were also significant differences between KS measure values at 30° of knee flexion with loads of 89, 111, and 133 N, and at 60° of knee flexion with a load of 133 N and ‘Advanced activities’ (KSS Functional Score subscale) (Table 3).

**Fig. 2:**
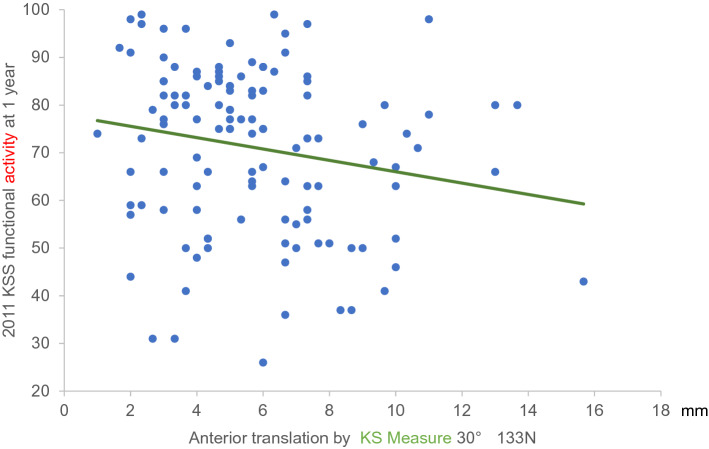
2011 KSS functional activities and anterior translation distance at KS measure 30° (133 N load). With the 2011 KSS functional activities on the *y*-axis and anterior translation distance at KS measure 30° based on a 133-N load on the *x*-axis, the equation of the regression line obtained was *y* = −1.2*x* + 77.9. *KSS* Knee Society Score

**Fig. 3:**
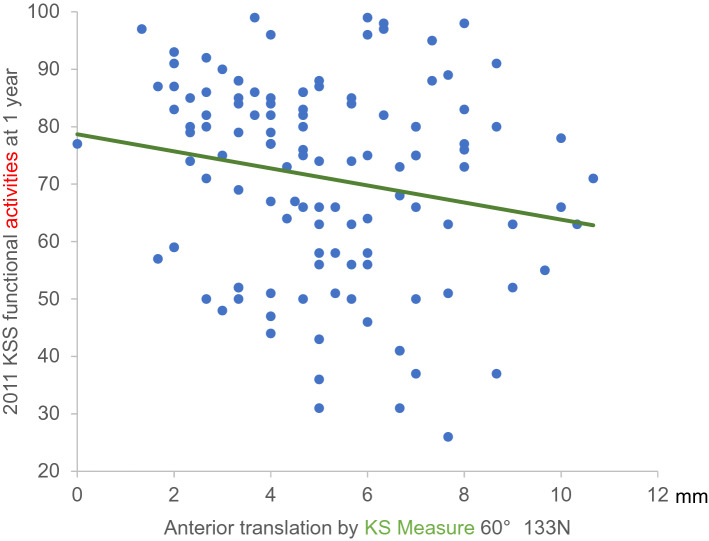
2011 KSS functional activities and anterior translation distance at KS measure 60° (133 N load). With KSS functional activities on the *y*-axis and anterior translation distance at KS measure 60° with a 133-N load on the *x*-axis, the equation of the regression line was y = −1.5x + 78.7. *KSS* Knee Society Score

Significant correlations between KSS functional activities and both anterior translation distance at KS measure 30° (133 N) and anterior translation distance at KS measure 60° (133 N) were also found (Figs. 2 and 3).

#### FJS

**Fig. 4:**
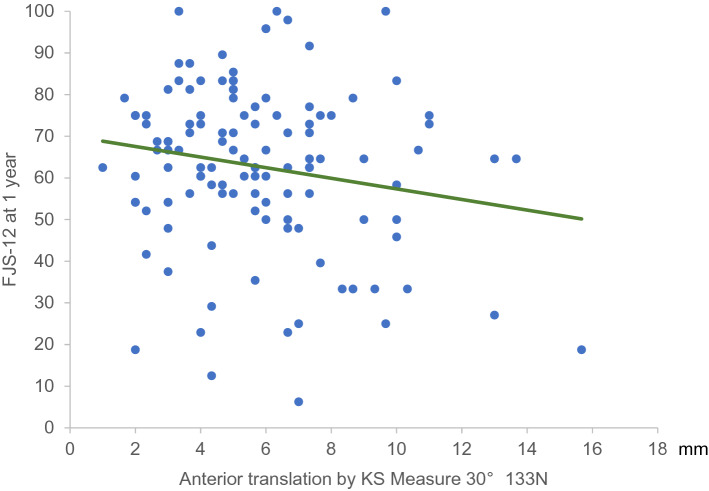
FJS-12 and anterior translation distance at KS measure 30° (133 N load) With FJS-12 on the *y*-axis and anterior translation by KS measure 30° with a 133 N load on the *x*-axis, the equation of the regression line obtained was *y* = −1.3*x* + 70.1. *KSS* Knee Society Score, *FJS* Forgotten Joint Score

Six months postoperatively, significant positive correlations were observed between KS measure values at 30° of knee flexion with loads of 89, 111, and 133 N, and at FJS9 and FJS10. One year postoperatively, there were significant positive correlations between all KS measure values and the FJS3 and FJS4 subitems of the FJS. Similarly, significant positive correlations were observed between KS measure values at 30° of knee flexion with loads of 111 and 133 N and FJS9. However, significant negative correlations were observed between KS measure values and the overall FJS score at 30° of knee flexion with a 133 N load (Table 3). A significant correlation between FJS12 and anterior translation distance by the KS measure at 30° of knee flexion with a 133 N load was observed (Fig. 4).

### Correlation between ROM and clinical scores at 1 year postoperatively

Knee extension angle was positively correlated with KSS parameters, including ‘Patient satisfaction’, ‘Patient expectations’, ‘Walking and standing’, ‘Standard activities’ (Functional score subscale), and ‘ROM’ (Objective knee indicator) (Table 4). In contrast, the knee extension angle was negatively correlated with FJS subitems, including FJS2, FJS3, FJS7, FJS9, FJS10, FJS11, and FJS12. However, the knee extension angle was positively correlated with the overall FJS score (Table 4).

**Table 4:** Correlations between ROM and KSS, FJS clinical scores at 1-year post-op (only items with significant correlations)

	KSS categories							FJS categories							
	Satisfaction	Expectations	IV Walking and standing	IV Standard activities	IV Advanced activities	ROM	Instability	2	3	7	9	10	11	12	Total
ROM extension															
Correlation co-efficient	0.30	0.20	0.19	0.24	0.16	0.72	−0.15	−0.18	−0.19	−0.23	−0.26	−0.21	−0.27	−0.24	0.22
* P* value (two-sided)	** < 0.01**	**0.03**	**0.04**	**0.01**	0.09	< 0.01	0.09	**0.05**	**0.04**	**0.01**	** < 0.01**	**0.02**	** < 0.01**	**0.01**	**0.01**
ROM flexion															
Correlation co-efficient	0.19	−0.14	0.12	0.33	0.25	0.61	−0.19	0.08	0.09	−0.17	−0.05	−0.04	−0.12	−0.09	0.04
* P* value (two-sided)	**0.04**	0.12	0.18	** < 0.01**	**0.01**	** < 0.01**	**0.03**	0.39	0.31	0.06	0.58	0.65	0.18	0.30	0.68

Cases with P<0.05 are shown in bold

*FJS* Forgotten joint score, *KSS* Knee Society Score, *Post-op* postoperative, *ROM *range of motion

Furthermore, the knee flexion angle was positively correlated with KSS parameters, including ‘Patient satisfaction’, ‘Standard activities’, ‘Advanced activities’ (Functional score subscales), and ‘ROM’ (Objective knee indicator). In contrast, the knee flexion angle was negatively correlated with ‘Instability’ (KSS Objective knee indicator) (Table 4). ROM was positively correlated with FJS for extension; however, ROM was not correlated with FJS for flexion (Table 4).

### Correlation between pain VAS and clinical scores at 1 year postoperatively

The pain VAS score was significantly correlated with several KSS parameters, including ‘Symptoms’, ‘Patient satisfaction’, ‘Walking and standing’, ‘Standard activities’, ‘Advanced activities’ (Functional score subscales), and ‘Alignment’ (Objective knee indicator) (Table 5). Similarly, the VAS pain score showed significant correlations with all 10 FJS subitems, as well as overall FJS score (Table 6).

**Table 5:** Correlations between pain and KSS clinical scores at 1-year post-op (only items with significant correlations)

	KSS categories					
	Symptoms	Satisfaction	IV Walking and standing	IV Standard activities	IV Advanced activities	Alignment
VAS pain						
Correlation co-efficient	−0.55	−0.37	−0.31	−0.39	−0.35	−0.19
*P* value (two-sided)	** < 0.01**	** < 0.01**	** < 0.01**	** < 0.01**	** < 0.01**	** < 0.05**

Cases with P<0.05 are shown in bold

*KSS *Knee Society Score, *Post-op* postoperative, *VAS* visual analogue scale

**Table 6:** Correlations between pain and FJS clinical scores at 1-year post-op (only items with significant correlations)

	FJS										
	2	3	4	5	7	8	9	10	11	12	Total
VAS pain											
Correlation co-efficient	0.29	0.30	0.20	0.29	0.23	0.19	0.34	0.28	0.42	0.34	−0.34
*P* value (two-sided)	** < 0.01**	** < 0.01**	** < 0.05**	** < 0.01**	** < 0.05**	** < 0.05**	** < 0.01**	** < 0.01**	** < 0.01**	** < 0.01**	** < 0.01**

Cases with P<0.05 are shown in bold

*FJS* Forgotten Joint Score, *Post-op *postoperative, *VAS* visual analogue scale

### Image assessment

The ICC (1, 1) for coronal alignment on 3D CT was perfect (CFA, 0.99; 95% confidence interval [CI] 0.98–0.99 and CTA, 0.99; 95% CI 0.97–0.99). The ICC (2, 1) for coronal alignment on 3D CT was also perfect, with a value of 0.83 (95% CI 0.65–0.94). The means, standard deviations, and *p* values of the unpaired t tests for image evaluations in the MP and PS groups were as follows: CFA (89.5° ± 1.9° vs. 89° ± 1.6°, *p* = 0.11); CTA (89° ± 1.7° vs. 89.6° ± 1.5°, *p* = 0.095); and HKAA (−1.4° ± 2.4° vs.−1.5° ± 2.1°, *p* = 0.95). The posterior tibial slope angle was 2.4° ± 1.7° in the MP group and 2.8° ± 1.9° in the PS group (*p* = 0.21). The posterior femoral condylar offset ratio was 0.52 ± 0.04 in the MP group and 0.52 ± 0.03 in the PS group (*p* = 0.51). The rotation angle of the femoral component was −0.7° ± 1.7° in the MP group and −1.0° ± 1.6° in the PS group (*p* = 0.32).

There were no significant differences in the imaging evaluation outcomes between the MP TKA and PS TKA groups using the unpaired *t *test.

### Adverse events

No adverse events, including infection, venous thromboembolism, fracture, revision, or death, occurred in either group. Moreover, there were no cases of laxity or clear zones in the radiographic frontal and lateral views at 1 year postoperatively in both groups.

## Discussion

The most important finding of this study was that MP TKA achieved better outcomes than PS TKA, which confirmed the hypothesis. The MP group showed significantly less translation at 60° of flexion than the PS group, whereas the translations of the two groups were similar at 30° of flexion. This finding may be attributed to the ball-and-socket joint design of the internal condyle of MP, which is highly constrained and advantageous with respect to AP stability; furthermore, MP compensates for both ACL and PCL functions. In the PS design, in contrast, the post-cam compensates for the PCL; however, its ACL compensation is weak. This suggests that MP TKA may be better than PS TKA for advanced activity, which correlates with the anterior translation distance at 60° knee flexion.

In this study, the MP group achieved better pain VAS, KSS, and FJS subitem scores than the PS group; differences in pain might also have influenced clinical scores given the strong correlations between VAS for pain and KSS and FJS [27]. The differences in the mean KSS values between the MP and PS groups were 2.4 for symptoms, 3.7 for satisfaction, and 8 for functional activity at 1 year postoperatively, which were much higher than the KSS minimum clinically important differences (MCIDs) of 1.9 for symptoms, 2.2 for satisfaction, and 4.1 for functional activities [27]. In particular, PS TKA is associated with patellar clunk syndrome, which causes a painful and palpable clunk when the knee moves from flexion to extension [10]. VAS in the PS group was associated with walking pain at approximately 30–40° of knee flexion, and this was suspected to be patellar clunk syndrome. CS-type TKA is less likely to cause patellar clunk syndrome. Dynamically, MP is considered more physiological than PS, and its movements may be associated with fewer symptoms, such as pain and discomfort.

Approximately 20% of patients are not satisfied after TKA [8]. In many TKA implant designs, the ACL is sacrificed, resulting in anterior instability, which may account for the dissatisfaction. Edelstein et al. [12] and Jones et al. [17] reported that sagittal stability was significantly higher in the MS group than in the PS group, and the MS group achieved significantly better clinical scores. Specifically, they observed a significant difference in the sagittal plane translation distance under stress at 30° of flexion between the MS (5.6 ± 1.9 mm) and PS (10.2 ± 2.7 mm) groups; however, the sagittal plane translation distance in the PS group differed from that in this study. The PS movement distance in the present study (133 N, 6.1 mm) was smaller than that reported by Edelstein et al., but was similar to that reported by Minoda et al. [26]. Consistently, Wautier et al. also found significantly higher sagittal plane stability in the MP group than in the PS group, although clinical scores were similar between the two groups [35]. Additionally, Samy et al. [30] and Batra et al. [4] reported that the MP group achieved significantly better clinical scores than the PS group. Nonetheless, other studies found no differences in clinical scores between these groups [3, 6, 11, 13, 21, 22, 32] and demonstrated poorer clinical results with the MP-type than with other implant designs [18, 19]. Therefore, the impact of the MP design requires further investigation. This study analysed a larger number of patients than these previous studies and focused on varus knee OA. Importantly, MP kinetics, which are generally considered to be physiological, may not apply to valgus knee OA.

The findings of the present study suggest that knee stability at 30° of flexion was advantageous for walking and advanced activities, with significantly better overall FJS scores. Specifically, the anterior translation distance at 30° of knee flexion (133 N) correlated more strongly with the FJS than the anterior translation at 60° of knee flexion with the same load. However, the anterior translation distance at 60° of knee flexion (133 N) was correlated with ‘Advanced Activities’ at 1 year postoperatively. Although a positive correlation was identified between anterior translation distance and knee flexion angle, knee stability may be prioritised over a greater flexion angle, especially considering that the knee flexion angle after TKA is most affected by the preoperative knee flexion angle. Correlations were found between KSS functional activities and the anterior translation distance at KS measure 30° (133 N), as well as anterior translation distance at KS measure 60° (133 N). Differences between the KSS scores were calculated by fitting the minimum and maximum values of the anterior translation distance at the KS measure 30° (133 N) and anterior translation distance at KS measure 60° (133 N) to the two regression lines (Figs. 2, 3). These differences were 17.6 and 16.5, respectively, which were much higher than the MCID of 4.1 for KSS functional activities [27]. In FJS12, the difference in FJS calculated by fitting the minimum-to-maximum values of the anterior translation distance at KS measure 30° (133 N) to the regression line (Fig. 4) was 19.1, which was higher than the MCID of 14 [14]. This suggests that anterior translation after TKA influenced the perceived differences in performance by patients.

Ishii et al. [15] measured AP laxity during a follow-up of ≥ 5 years after TKA and reported that the best postoperative results were obtained when AP laxity was ≤ 6 mm. Consistently, the present study showed that a shorter anterior translation distance was associated with better postoperative function. However, AP laxity and postoperative pain were not correlated in the present study, which contrasts with the findings of Matsumoto et al. [24] who reported that postoperative AP laxity at 60° of knee flexion was significantly correlated with patient-reported pain.

Regarding ROM, postoperative restriction of knee extension was correlated with poor clinical outcomes in walking and basic activities in the KSS and most FJS items; therefore, full extension should be achieved postoperatively. Although knee flexion angle was not correlated with FJS, it was correlated with the KSS parameters ‘Patient satisfaction’, ‘Standard activities’, and ‘Advanced activities’. Many of the KSS parameter scores and FJS scores were correlated with pain VAS score, suggesting that residual pain after TKA directly affects surgical outcome, and that identifying and improving these related factors should be prioritised.

This study has some limitations. First, this was not a randomised controlled trial, and surgeons differed between the comparison groups due to their preferences for particular implant systems. However, the two surgeons were equally experienced, and the surgical technique was consistent. One was the lead surgeon, while the other assisted, and vice versa, for the two groups; thus, there should not have been any major intersurgeon variabilities. No significant differences were found between the two groups in alignment evaluation by radiography or CT. Second, the evaluation of the anterior translation distance was not performed with loading. In MP, weight loading may increase the stability of the ball joint of the condyle, which may be more advantageous to AP stability under loading. Third, sex and preoperative extension angle differed significantly between groups. Preoperative extension was significantly more restricted in the MP group; however, it was similar in the groups postoperatively. Therefore, extension may not have affected clinical outcomes. Although there were fewer men in the PS group than in the MP group, women achieved at least the same level of functional improvement as men [28]. Furthermore, because the preoperative scores were similar, it is unlikely that sex differences affected the clinical results in the comparisons. Finally, the study only reported short-term findings. Cacciola et al. [9] reported that the mean 8-year survival rate for MP TKA was 97.6%, which was comparable to those of other standard implants. Bordini et al. [7] reported that the 10-year survival rate for MP TKA was 96.3%, which was comparable to other cemented TKA systems. However, another registry reported lower survival rates for MP, suggesting that careful follow-up is required [29].

## Conclusions

In TKA for varus knee OA, MP TKA significantly improved postoperative function and pain compared with PS TKA. Since anterior translation distance and postoperative pain were not correlated, factors other than implant design and unrelated to AP stability might be responsible for the reduced pain in MP TKA compared with that in PS TKA.

## Supplementary Information

Below is the link to the electronic supplementary material.

Supplementary file1 (XLSX 327 KB)

## Data Availability

The data that support the findings of this study are available from the corresponding author, MK, upon reasonable request.

## Abbreviations

ACL: Anterior cruciate ligament
AP: Anterior–posterior
CI: Confidence interval
CFA: Coronal femoral component angle
CS: Cruciate substitute
CT: Computed tomography
CTA: Coronal tibial component angle
FJS: Forgotten Joint Score
HKAA: Hip–knee–ankle angle
ICC: Intraclass correlation co-efficient
KSS: Knee Society Score
MCID: Minimum clinically important difference
MP: Medial pivot
OA: Osteoarthritis
PCL: Posterior cruciate ligament
PS: Posterior-stabilised
ROM: Range of motion
TKA: Total knee arthroplasty
VAS: Visual analogue scale

## References

[1] Abdelnasser MK, Elsherif ME, Bakr H, Mahran M, Othman MHM, Khalifa Y (2019). All types of component malrotation affect the early patient-reported outcome measures after total knee arthroplasty. Knee Surg Relat Res.

[2] Akagi M, Mori S, Nishimura S, Nishimura A, Asano T, Hamanishi C (2005). Variability of extraarticular tibial rotation references for total knee arthroplasty. Clin Orthop Relat Res.

[3] Bae DK, Cho SD, Im SK, Song SJ (2016). Comparison of midterm clinical and radiographic results between total knee arthroplasties using medial pivot and posterior-stabilized prosthesis-a matched pair analysis. J Arthroplast.

[4] Batra S, Malhotra R, Kumar V, Srivastava DN, Backstein D, Pandit H (2021). Superior patient satisfaction in medial pivot as compared to posterior-stabilized total knee arthroplasty: a prospective randomized study. Knee Surg Sports Traumatol Arthrosc.

[5] Behrend H, Giesinger K, Giesinger JM, Kuster MS (2012). The “forgotten joint” as the ultimate goal in joint arthroplasty: validation of a new patient-reported outcome measure. J Arthroplast.

[6] Benjamin B, Pietrzak JRT, Tahmassebi J, Haddad FS (2018). A functional comparison of medial pivot and condylar knee designs based on patient outcomes and parameters of gait. Bone Jt J.

[7] Bordini B, Ancarani C, Fitch DA (2016). Long-term survivorship of a medial-pivot total knee system compared with other cemented designs in an arthroplasty registry. J Orthop Surg Res.

[8] Bourne RB, Chesworth BM, Davis AM, Mahomed NN, Charron KDJ (2010). Patient satisfaction after total knee arthroplasty: who is satisfied and who is not?. Clin Orthop Relat Res.

[9] Cacciola G, Mancino F, De Meo F, Di Matteo V, Sculco PK, Cavaliere P (2021). Mid-term survivorship and clinical outcomes of the medial stabilized systems in primary total knee arthroplasty: a systematic review. J Orthop.

[10] Choi WC, Ryu KJ, Lee S, Seong SC, Lee MC (2013). Painful patellar clunk or crepitation of contemporary knee prostheses. Clin Orthop Relat Res.

[11] Dowsey MM, Gould DJ, Spelman T, Pandy MG, Choong PF (2020). A randomized controlled trial comparing a medial stabilized total knee prosthesis to a cruciate retaining and posterior-stabilized design: a report of the clinical and functional outcomes following total knee replacement. J Arthroplast.

[12] Edelstein AI, Bhatt S, Wright-Chisem J, Sullivan R, Beal M, Manning DW (2020). The effect of implant design on sagittal plane stability: a randomized trial of medial- versus posterior-stabilized total knee arthroplasty. J Knee Surg.

[13] Gray HA, Guan S, Young TJ, Dowsey MM, Choong PF, Pandy MG (2020). Comparison of posterior-stabilized, cruciate-retaining, and medial-stabilized knee implant motion during gait. J Orthop Res.

[14] Ingelsrud LH, Roos EM, Terluin B, Gromov K, Husted H, Troelsen A (2018). Minimal important change values for the Oxford Knee Score and the Forgotten Joint Score at 1 year after total knee replacement. Acta Orthop.

[15] Ishii Y, Matsuda Y, Ishii R, Sakata S, Omori G (2005). Sagittal laxity in vivo after total knee arthroplasty. Arch Orthop Trauma Surg.

[16] Jacobs CA, Christensen CP, Karthikeyan T (2016). An intact anterior cruciate ligament at the time of posterior cruciate ligament-retaining total knee arthroplasty was associated with reduced patient satisfaction and inferior pain and stair function. J Arthroplast.

[17] Jones CW, Jacobs H, Shumborski S, Talbot S, Redgment A, Brighton R (2020). Sagittal stability and implant design affect patient reported outcomes after total knee arthroplasty. J Arthroplast.

[18] Kim YH, Park JW, Kim JS (2017). Clinical outcome of medial pivot compared with press-fit condylar sigma cruciate-retaining mobile-bearing total knee arthroplasty. J Arthroplast.

[19] Kim YH, Yoon SH, Kim JS (2009). Early outcome of TKA with a medial pivot fixed-bearing prosthesis is worse than with a PFC mobile-bearing prosthesis. Clin Orthop Relat Res.

[20] Landis JR, Koch GG (1977). The measurement of observer agreement for categorical data. Biometrics.

[21] Lee QJ, Wai Yee EC, Wong YC (2020). No difference in patient preference for medial pivot versus posterior-stabilized design in staged bilateral total knee arthroplasty: a prospective study. Knee Surg Sports Traumatol Arthrosc.

[22] Lin Y, Chen X, Li L, Li Z, Zhang Y, Fan P (2020). Comparison of patient satisfaction between medial pivot prostheses and posterior-stabilized prostheses in total knee arthroplasty. Orthop Surg.

[23] Matsuda S, Kawahara S, Okazaki K, Tashiro Y, Iwamoto Y (2013). Postoperative alignment and ROM affect patient satisfaction after TKA. Clin Orthop Relat Res.

[24] Matsumoto K, Ogawa H, Yoshioka H, Akiyama H (2017). Postoperative anteroposterior laxity influences subjective outcome after total knee arthroplasty. J Arthroplast.

[25] Matsumoto M, Baba T, Homma Y, Kobayashi H, Ochi H, Yuasa T (2015). Validation study of the Forgotten Joint Score-12 as a universal patient-reported outcome measure. Eur J Orthop Surg Traumatol.

[26] Minoda Y, Ikebuchi M, Mizokawa S, Ohta Y, Nakamura H (2016). Mobile-bearing TKA improved the anteroposterior joint stability in mid-flexion range comparing to fixed-bearing TKA. Arch Orthop Trauma Surg.

[27] Nishitani K, Yamamoto Y, Furu M, Kuriyama S, Nakamura S, Ito H (2019). The minimum clinically important difference for the Japanese version of the new Knee Society Score (2011KSS) after total knee arthroplasty. J Orthop Sci.

[28] O’Connor MI (2011). Implant survival, knee function, and pain relief after TKA: are there differences between men and women?. Clin Orthop Relat Res.

[29] Øhrn FD, Gøthesen Ø, Låstad Lygre SH, Peng Y, Lian ØB, Lewis PL (2020). Decreased survival of medial pivot designs compared with cruciate-retaining designs in TKA without patellar resurfacing. Clin Orthop Relat Res.

[30] Samy DA, Wolfstadt JI, Vaidee I, Backstein DJ (2018). A retrospective comparison of a medial pivot and posterior-stabilized total knee arthroplasty with respect to patient-reported and radiographic outcomes. J Arthroplast.

[31] Scuderi GR, Bourne RB, Noble PC, Benjamin JB, Lonner JH, Scott WN (2012). The new Knee Society Knee Scoring system. Clin Orthop Relat Res.

[32] Shi W, Jiang Y, Wang C, Zhang H, Wang Y, Li T (2020). Comparative study on mid- and long-term clinical effects of medial pivot prosthesis and posterior-stabilized prosthesis after total knee arthroplasty. J Orthop Surg Res.

[33] Taniguchi N, Matsuda S, Kawaguchi T, Tabara Y, Ikezoe T, Tsuboyama T (2015). The KSS 2011 reflects symptoms, physical activities, and radiographic grades in a Japanese population. Clin Orthop Relat Res.

[34] Tsukiyama H, Kuriyama S, Kobayashi M, Nakamura S, Furu M, Ito H (2017). Medial rather than lateral knee instability correlates with inferior patient satisfaction and knee function after total knee arthroplasty. Knee.

[35] Wautier D, Thienpont E (2017). Changes in anteroposterior stability and proprioception after different types of knee arthroplasty. Knee Surg Sports Traumatol Arthrosc.

